# *Nephromyces* Encodes a Urate Metabolism Pathway and Predicted Peroxisomes, Demonstrating That These Are Not Ancient Losses of Apicomplexans

**DOI:** 10.1093/gbe/evy251

**Published:** 2018-11-30

**Authors:** Christopher Paight, Claudio H Slamovits, Mary Beth Saffo, Christopher E Lane

**Affiliations:** 1 Department of Biological Sciences, University of Rhode Island; 2 Department of Biochemistry and Molecular Biology, Dalhousie University, Halifax, Nova Scotia, Canada; 3 Smithsonian National Museum of Natural History, Washington, District of Columbia

**Keywords:** apicomplexan, tunicates, peroxisomes, purine degradation, *Nephromyces*, *Cardiosporidium*

## Abstract

The phylum Apicomplexa is a quintessentially parasitic lineage, whose members infect a broad range of animals. One exception to this may be the apicomplexan genus *Nephromyces*, which has been described as having a mutualistic relationship with its host. Here we analyze transcriptome data from *Nephromyces* and its parasitic sister taxon, *Cardiosporidium*, revealing an ancestral purine degradation pathway thought to have been lost early in apicomplexan evolution. The predicted localization of many of the purine degradation enzymes to peroxisomes, and the in silico identification of a full set of peroxisome proteins, indicates that loss of both features in other apicomplexans occurred multiple times. The degradation of purines is thought to play a key role in the unusual relationship between *Nephromyces* and its host. Transcriptome data confirm previous biochemical results of a functional pathway for the utilization of uric acid as a primary nitrogen source for this unusual apicomplexan.

## Introduction

Apicomplexans are most well known for being parasites of humans and livestock. Species in the genus *Plasmodium*, for instance, are the etiological agents of malaria. Apicomplexan species show tremendous variation in transmission methods, life cycles, host range, host manipulation strategies, cell-types infected, metabolic capabilities, immune evasion strategies, and virulence (Roos 2005; Reid et al. 2012; Kemp et al. 2013; Cardoso et al. 2016). Because of this variability, there are few apicomplexan characteristics shared throughout the phylum. Among the few universal apicomplexan features are a parasitic life history and an inability to degrade purines (Janouskovec and Keeling 2016). *Nephromyces*, a derived apicomplexan genus of uncertain phylogenetic placement, appears to be an exception to both of these traits.

*Nephromyces* was misclassified as a fungus for more than a 100 years, based on long hyphal-like cell structures, flagellated spores interpreted by some as chytrid zoospores and cell walls made of a chitin (Giard 1888). It was not until the application of molecular methods that *Nephromyces* was confirmed as a member of the derived apicomplexans (Saffo et al. 2010). Although some analyses have tentatively placed it sister to adeleid coccidia, or piroplasms, the precise phylogenetic position of *Nephromyces* remains unresolved (Saffo et al. 2010; Janouškovec et al. 2015). *Nephromyces* species are monoxenous (infecting a single host) and are found exclusively in the Molgulidae family of tunicates (Saffo and Davis 1982). In a phylum composed of obligate parasites, the feature that distinguishes *Nephromyces* is its apparent mutualistic relationship with its tunicate hosts. The mutualistic relationship has been inferred based primarily on the nearly 100% infection rate and lack of clearance from the host (Saffo and lowenstam 1978, 1988; saffo and davis 1982; Saffo et al. 2010). We use this label with caution, given how complex host–symbiont dynamics can be, how the costs and benefits of both “harmful” and “beneficial” symbioses can be difficult to determine, and how they can vary with genomic changes in hosts and symbionts (Leung and Poulin 2008; Saffo 2014; Mushegian and Ebert 2016).

A shift in lifestyle from obligate parasite to mutualistic symbiont is quite rare, and completely unknown from deep within a eukaryotic lineage with such a long evolutionary history of parasitism. One common consequence of a parasitic lifestyle is a loss of genes essential to free-living organisms (Greganova et al. 2013; Janouškovec et al. 2015; Petersen et al. 2015; Zarowiecki and Berriman 2015). In an intracellular environment, if precursor molecules can be scavenged, there is less selective pressure to maintain biosynthesis pathways, and many are consequently lost (Keeling 2004; Sakharkar et al. 2004; Morrison et al. 2007). In phyla such as Apicomplexa, these losses can be extreme and over half of the genes found in their photosynthetic sister group, chromerids, have been lost in apicomplexans (Woo et al. 2015).

With so many basic metabolic functions lost, and with such dependence on the host, it is difficult to see how the relationship between host and parasite could change to a mutualistic interaction. However, one way for an organism to rapidly change its metabolic capabilities is to take on a bacterial symbiont. *Nephromyces* has done just that, leading to the hypothesis that bacterial endosymbionts inside *Nephromyces* perform some of the metabolic functions lost in Apicomplexa, and potentially contribute something beneficial to the tunicate host (Saffo 1990; Saffo et al. 2010). Bacterial endosymbionts are common across the tree of life (although rare in apicomplexans) and perform a wide variety of functions for their hosts (Nowack and Melkonian 2010). These include amino acid metabolism and vitamin metabolism (Moran et al. 2005), nitrogen metabolism (Lopez-Sanchez et al. 2009), defense (De Souza et al. 2009), chemotrophic energy production (Urakawa et al. 2005), and photosynthesis (Marin et al. 2005), to name a few.

A tempting hypothesis for the functional role of *Nephromyces* bacterial endosymbionts is the breakdown of purines to urea in the purine degradation pathway (Saffo 1990). In support of this hypothesis *Nephromyces-*infected tunicates have quite high levels of the enzyme urate oxidase, which catalyzes conversion of uric acid to 5-hydroxyisourate, but the enzyme is undetectable in uninfected tunicates (Mahler et al. 1955; Saffo 1988). Coupled with the fact that all known apicomplexans and tunicates have lost the purine degradation pathway, these data were suggestive of a bacterial contribution to purine degradation.

In a yet-unexplained quirk of tunicate biology, many tunicate species have localized deposits of uric acid (Goodbody 1965; Saffo and Lowenstam 1978; Lambert et al. 1998). Storage as a form of excretion, nitrogen storage for future release, and structural support are among the proposed functions of tunicate urate deposits (Goodbody 1965; Saffo 1988; Lambert et al. 1998). Tunicates in the Molgulidae family have the largest uric acid deposits, which are localized to a specialized, ductless structure, called a renal sac (Saffo and Lowenstam 1978). These uric acid deposits occur regardless of infection status, indicating a tunicate origin of these purine deposits. Despite the name, the renal sac has many features (most notably, the absence of any ducts or macroscopic openings) atypical for an excretory organ, and its biological function has yet to be determined. *Nephromyces* infects feeding molgulid tunicates after the postmetamorphic onset of host feeding and completes its entire lifecycle within the renal sac. Four factors led to the conclusion that the bacterial endosymbionts within *Nephromyces* are the source of urate oxidase activity in this system: 1) the colonization of *Nephromyces* within a structure with high concentrations of urate, 2) the absence of urate oxidase activity in the molgulid hosts (Saffo 1988, 1991), 3) the high urate oxidase activity found in *Nephromyces* (including its bacterial symbionts: Saffo 1988, 1991), coupled with 4) the lack of obvious ultrastructural evidence of peroxisomes in *Nephromyces* (Saffo 1990).

It is logical to think that the addition of bacterial endosymbionts to *Nephromyces* might have been key to colonizing this novel purine-rich niche, and is how *Nephromyces* escaped the “evolutionary dead end” of a parasitic lifestyle. In order to test this directly, and examine the metabolic relationships between the tunicate host, *Nephromyces*, and its bacterial endosymbionts, we sequenced the community transcriptome. To identify possible evolutionary or physiological changes involved in coevolution of *Nephromyces* with its molgulid hosts, we also sequenced the transcriptome of a sister taxon of *Nephromyces*, *Cardiosporidium cionae* (Ciancio et al. 2008; Saffo et al. 2010), an apicomplexan parasite found in the blood in a broad range of nonmolgulid ascidian hosts, including *Ciona intestinalis, Styela clava, Halocynthia roretzi*, and *Ascidiella aspersa* (Dong et al. 2006; Ciancio et al. 2008)*.* Interestingly, *Cardiosporidium cionae* also harbors bacterial endosymbionts, which allows for a more direct comparison between *Nephromyces* and *Cardiosporidium*.

Here we confirm the exceptionally high levels of urate oxidase activity in tunicates with *Nephromyces*, and extend this result to include high expression levels of all the genes in the purine degradation pathway (xanthine dehydrogenase, urate oxidase, 5-hydroxyisourate hydrolase, 2-oxo-4-hydroxy-4-carboxy-5-ureidoimidazoline decarboxylase, and allantoinase). The breakdown of purines starts by conversion to xanthine. Xanthine then enters the ureide pathway and the enzyme xanthine dehydrogenase catalyzes the reaction of xanthine to urate (Xi et al. 2000; Nishino et al. 2008). Urate oxidase catalyzes the oxidation of uric acid to 5-hydroxyisourate. Following conversion the enzyme 5-hydroxyisourate hydrolase catalyzes 5-hydroxyisourate to 5-hydroxy-2-oxo-4-ureido-2,5-dihydro-1H-imidazole-5-carboxylate (Kahn and Tipton 1998). This is further processed into (s)-allantoin by the enzyme 2-oxo-4-hydroxy-4-carboxy-5-ureidoimidazoline decarboxylase (Jung et al. 2006). Allantoinase catalyzes (s)-allantoin into allantoate. From this point, there are a few different pathways with different end points that organisms are able to shuttle allantoate to (Cusa et al. 1999). A common end point is to process allantoate into urea and ureidoglycolate, to be further converted into carbon dioxide and ammonia. Alternatively, ureidoglycolate can be converted to glyoxylate, or the urea may be excreted as waste (Schultz et al. 2001; Werner et al. 2010).

We confirm that all the genes necessary for purine degradation are encoded by *Nephromyces* and *Cardiosporidium*, and not their endosymbiotic bacteria. Although the expression of urate oxidase by *Nephromyces* and *Cardiosporidium* is unexpected, a parallel issue is where the urate oxidase is physically located in the cell, given that apicomplexans reportedly lack peroxisomes (Schluter et al. 2006). Urate oxidase activity is restricted to peroxisomes in eukaryotes, due to the numerous toxic byproducts that are produced in the breakdown of uric acid. Research into peroxisomes in Apicomplexa has a complex and contradictory history, with studies reporting both the presence (Kaasch and Joiner 2000; Gabaldon et al. 2016) and absence (Ding et al. 2000; Schluter et al. 2006; Gabaldon 2010) of peroxisomes in Apicomplexa. Recent work by Moog et al. (2017) and Ludewig-Klingner et al. (2018) demonstrates compelling support for peroxisomes in coccidians. Both studies present comprehensive bioinformatic (and also proteomic, in part) evidence for the presence of peroxisomal biogenesis factors (peroxins) and typical peroxisomal metabolic enzymes (including predicted relevant targeting signals) in coccidians (Moog et al. 2017; Ludewig-Klingner et al. 2018). However, neither article provides explicit experimental evidence (e.g., microscopic) for the formation of peroxisomes in these organisms. Although direct evidence is still absent, both studies point to Lige et al. (2009) and their identification of peroxisome-like vesicles in *Toxoplasmagondii*, for possible microscopic support.

Our data demonstrate that *Nephromyces* encodes a complete purine degradation pathway and a number of proteins predicted to be targeted to, or involved in, peroxisome biogenesis, maintenance and protein import, providing novel support of peroxisomes in Apicomplexa. Additionally, we propose the functional significance of purine degradation in *Nephromyces*, and reject the hypothesis that bacterial endosymbionts facilitated an escape from parasitism by providing genes in the purine degradation pathway.

## Materials and Methods

### *Molgula m* *anhattensis* Collection and Lab Culture

*Molgula manhattensis* tunicates were collected from a dock in Greenwich Bay, RI (41°39′22.7″N, 71°26′53.9″W) on July 2014. For transcriptomic analysis, a single renal sac was separated from one tunicate, and all extraneous tissue removed. The intact renal sac was placed in liquid nitrogen for 5 min and then stored at −80 °C for later RNA extraction. Gonads were dissected from five, sexually mature, *M. manhattensis*, collected from the same population in Greenwich Bay, RI on August 2014. Eggs and sperm were mixed with sterile seawater and divided evenly between two petri dishes. Plates were incubated at room temperature for 2 days with daily 100% water changes. Tunicate larvae attached to the bottom and sides of the petri dishes by day 3. By day 4, larvae had metamorphosed into adults and were actively feeding. Plates were moved to an incubator at 18 °C with a 24-h dark cycle to limit growth of contaminants. Tunicates were fed by 100% water exchange with cultures of *Isochrysis galbana* and *Chaetoceros gracilis* 3 days a week. After several weeks, tunicates were moved to aerated beakers to meet their increased nutrient and gas exchange requirements. Feeding regimen remained the same except that food volume was increased with tunicate growth. Tunicates were grown for 6 months until they were ∼10 mm across. Each renal sac was placed into a 1.5-ml Eppendorf tube and flash frozen in liquid nitrogen. Polymerase chain reaction screens confirmed that *Nephromyces* was absent from lab-raised individuals. Lab-grown tunicates were split into two groups. Renal sacs were harvested from three tunicates to use as transcriptome controls. A second group was infected with *Nephromyces* oocysts to limit coinfections from multiple species and raised for genomic analysis.

### *Cardiosporidium c* *ionae* Collection, Isolation, and Concentration

*Ciona intestinalis* were collected from docks in Snug Harbor, RI (41.3890°N, 71.5201°W), on August 2017. Tunics were removed and the body wall was opened to allow access to the heart. A sterile syringe was used to remove cardiac blood as cleanly as possible. Blood was kept at 4 °C until *Cardiosporidium* infection was verified using Giemsa stain to visualize *Cardiosporidium.* Heavily infected samples were pooled together and centrifuged at 500 × g for 5 min. The resulting supernatant was removed, and the samples were frozen in liquid nitrogen and stored at −80 °C. Samples with low rates of infection were enriched for *Cardiosporidium* using sucrose gradients (Arrowood and Sterling 1987; Ogedengbe et al. 2015). Gradients of 20%, 25%, 30%, 35%, and 40% sucrose solutions in phosphate buffer were layered together. Approximately 5 ml of tunicate blood was added to the column and centrifuged at 500 × g for 30 min at 4 °C. The 25% and 30% layers were collected (based on visual screens showing high *Cardiosporidium* cell density and low tunicate cell density), washed in phosphate-buffered saline, pelleted and then frozen in liquid nitrogen and stored at −80 °C.

### RNA Extraction

RNA extraction buffer (Zymo Reasearh LLC, Irvine, CA) was added to samples and ground with a pestle. Following grinding, the Zymo Quick-RNA kit (Zymo Research LLC) was used and the manufacturer’s protocol was followed. RNA was converted to cDNA and sequenced at the School of Medicine Genome Resource Center, University of Maryland. Five separate paired-end RNA libraries (two from infected renal sac and three from uninfected renal sac) were multiplexed on one lane of the Illumina HiSeq platform, resulting in 326,299,923, 327,957,761, and 316,754,780 reads for the three renal sacs without *Nephromyces*, and 40,606,230 from the wild *M. manhattensis* renal sac. For *Cardiosporidium*, three samples of *C. intestinalis* blood were used: One with unseparated blood, one enriched with cells collected at the 25% sucrose gradient, and one enriched with cells from the 30% sucrose gradient were multiplexed on one lane of the Illumina HiSeq platform, resulting in 92,250,706, 109,023,104, and 110,243,954, respectively. Transcriptome data were assembled and proteins were predicted with the Trinity/Trinotate pipeline version 2.4.0 run on the server at Brown University Center for Computation and Visualization (Haas et al. 2014). Reads assembled into 115,457, 388,535, and 109,446 contigs from infected, uninfected samples, and *C. intestinalis*, respectively. Protein sequences were predicted using Transdecoder (Haas et al. 2014). Transcriptome completeness was assessed with Busco v3 against the Eukaryotic reference data sets (Simão et al. 2015).

### DNA Extraction

The renal sacs from eight lab-grown *M. manhattensis* individuals were dissected, and their renal fluid was pooled in a 1.5-ml Eppendorf tube. Contents were centrifuged at 8,000 × g for 5 min to pellet *Nephromyces* cells, and following centrifugation the renal fluid was discarded. Five hundred microliters of CTAB buffer with 5 μl of proteinase K and ceramic beads were added to the pelleted *Nephromyces* cells. The sample was placed in a bead beater for 3 min and then on a rotator for 1.5 h at room temperature. Five hundred microliters of chloroform was added, mixed gently, and centrifuged for 5 min. The top layer was removed, and 2× the sample volume of ice-cold 100% EtOH and 10% sample volume of 3 M sodium acetate were added to the sample and incubated at −20 °C overnight. The sample was centrifuged at 16,000 × g for 30 min and the liquid was removed. Ice-cold 70% EtOH was added and centrifuged at 16,000 × g for 15 min. Liquid was removed and sample air dried for 2 min. DNA was re-eluted in 50 μl of deionized water.

### Illumina Sequencing

A nanodrop (2000c, ThermoScientific) was used to assess DNA purity and DNA concentration, and an agarose gel was run to assess genomic DNA fragmentation. Following quality control, an Illumina library was constructed. Library preparation and sequencing were done at the URI Genomics and Sequencing Center (URIGSC). The completed library was sequenced on the Illumina MiSeq platform at the URIGSC and the HiSeq platform at the University of Baltimore sequencing center on three lanes.

### Pacific Biosciences Sequencing

Using the contents of 150 (done in batches of 10 then pooled) *M. manhattensis* renal sacs, the same DNA extraction protocol was performed as for Illumina sequencing. DNA was sequenced using three SMRT cells on the Pacific Biosciences platform at the University of Baltimore sequencing center.

### Illumina Sequence Data Assembly

One MiSeq lane and three lanes of HiSeq, all from the same library, were trimmed using Trimmomatic (Bolger et al. 2014) and then assembled using Spades assembler (Bankevich et al. 2012) on the URI server BlueWaves.

### Pacific Biosciences Sequence Data Assembly

Pacific Biosciences reads were error corrected using pbsuite/15.8.24 (English et al. 2012) on the Brown University server, Oscar. Reads were then assembled using Canu (Koren et al. 2017). Contigs generated by Canu were combined with Illumina MiSeq/HiSeq short reads with Abyss v2.02 (Jackman et al. 2017).

### Sequence Annotation

Genes in the urate pathway were identified initially using KEGG GhostKOALA and KASS and subsequently by BlastP searches against NCBI’s nr protein database (Kanehisa et al. 2016). All candidate genes were screened using InterProScan to predict function (Finn et al. 2017). A curated database of phylogenetically representative species with good quality annotations for the three purine degradation genes and malate synthase (MLS) were downloaded from NCBI. These genes were then used to construct gene trees.

Sequences were aligned with MAFFT (Katoh and Standley 2013) using FFT-NS-i. Maximum-likelihood phylogenetic trees were constructed and performed with RAxML (Stamatakis 2014) using the GAMMA model with 1,000 seed trees and 1,000 bootstrap replicates. Trees were viewed and modified using Figtree (v1.4.0, http://tree.bio.ed.ac.uk/software/figtree/; last accessed December 19, 2018).

Protein sequences were used to search against PeroxisomeDB (Schlüter et al. 2009), and BLAST hits lower than e^−20^ were retained and used in a BlastP query against NCBI’s Refseq protein database (Schlüter et al. 2009). Additional peroxisomal genes were identified with KAAS (Moriya et al. 2007). As many of these peroxisome genes are encoded by *M. manhattensis*, all copies that had a closest hit to opisthokonts or bacteria were removed. Transcripts from uninfected *M. manhattensis* were used to screen additional tunicate genes using cd-hit at a 90% identity level (Li and Godzik 2006). Remaining genes were tested for signal motifs and subcellular location predictions with Wolf PSORTII, Ppero, TargetP, topcons, and Predotar (supplementary table 1, Supplementary Material online) (Nakaia and Horton 1999; Small et al. 2004; Emanuelsson et al. 2007; Bernsel et al. 2009; Wang et al. 2017).

*Nephromyces*-specific RNAseq reads were mapped to our genomic assembly using bowtie2 (Langmead and Salzberg 2012) with the –verysensitive flag set. Following mapping, Bedtools (Quinlan and Hall 2010) was used to quantify coverage across contigs, which were separated based on coverage levels. Contigs identified as *Nephromyces* were annotated using Maker2 with ab initio gene predictions from Augustus (Stanke et al. 2004; Holt and Yandell 2011).

## Results

The contents of a single renal sac from an individual *M.manhattensis* resulted in 195,694 transcripts from *M. manhattensis*, *Nephromyces*, and the bacterial endosymbionts. After binning by species, 60,223 transcripts were attributed to *Nephromyces*. The cardiac fluid from 40 infected *Ciona intestinalis* individuals resulted in 109,446 transcripts, including 15,541 *Cardiosporidium* transcripts. The BUSCO algorithm was used to assess the completeness of the transcriptomes and reported 81.8% complete transcripts and 6.3% partial for the *Nephromyces* data and 69.7% complete and 11.9% partial for *Cardiosporidium*.

**Fig. 1.**
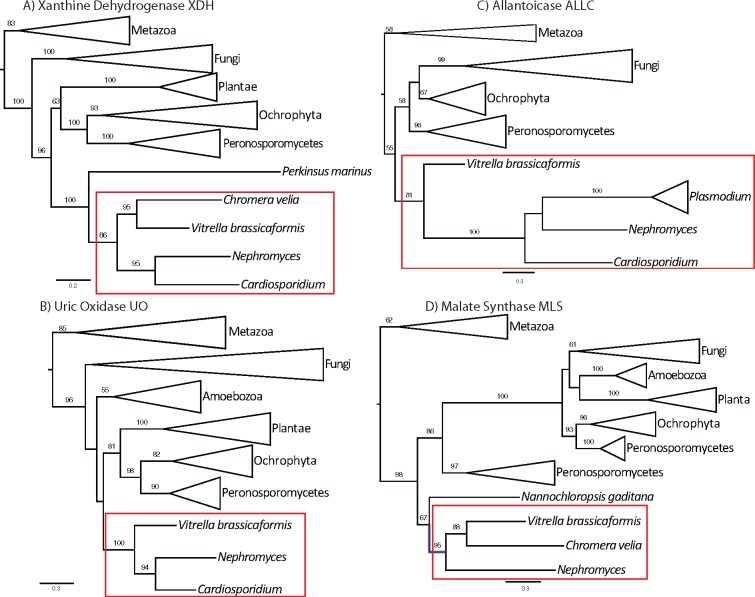
—Maximum-likelihood protein trees of (*A*) xanthine dehydrogenase, (*B*) urate oxidase, (*C*) allantoicase, and (*D*) MLS. Genes (*A*–*C*) are involved in purine degradation and their position supports an ancestral apicomplexan purine degradation pathway in *Nephromyces/Cardiosporidium.* MLS (*D*) acts on glyoxylate and acetyl-CoA to produce malate to complete the pathway. Stramenopiles are paraphyletic in the MLS phylogeny, possibly indicating a deep gene duplication. Although the support for deeper nodes is variable among all four genes, there is consistent support for a monophyletic origin of *Nephromyces/Cardiosporidium* genes with Chromerids (red box). Major lineages have been collapsed for presentation. Support values are percentage bootstrap support above 50%.

The *Nephromyces* genome assembly consists of 1,176 contigs >5 kb with a maximum length of 287,191 bp and an average length of 36 kb (Paight et al., in preparation). This data set was used to search for purine degradation genes to determine their genomic context. All of the purine degradation genes, as well as *MLS*, were predicted and annotated in the genome by Maker2. All genes but *URAD* contained introns, and neighboring genes on the identified contigs had top BLAST hits to apicomplexans in all cases (table 1), indicating that they are encoded in the *Nephromyces* genome, not the endosymbiotic bacteria or host *M.manhattensis*. Phylogenetic trees for xanthine dehydrogenase, uric oxidase, MLS, and allantoicase consistently resolve the monophyly of *Nephromyces, Cardiosporidium*, and Chromerids (fig. 1). Chromerids are the photosynthetic and the closest free-living relatives of Apicomplexa (Moore et al. 2008), indicating a vertical inheritance of this pathway from the common ancestor of apicomplexans.

**Table 1:** Genomic Context of the Annotated Purine Degradation Genes and MLS, in the *Nephromyces* Genomic Assembly

Gene	Introns in Gene	Contig	Contig Length (kb)	Predicted Genes on Contig	Genes with Top Apicomplexan BLAST Hits
XDH	4	Neph_3686418	24.5	4	2
UO	7	Neph_3687015	82.5	12	7
uraH	2	Neph_3685393	94.3	6	4
URAD	0	Neph_3687674	30.9	6	5
ALLC	10	Neph_3687655	116.3	16	11
MS	6	Neph_3671841	7	2	1

Note.—The phylogenetic affiliation of neighboring genes on each contig was identified by top hit against the NCBI nr database using BlastP. Every contig encoding a target gene included other apicomplexan genes, and genes that did not hit apicomplexans had no strong affinity for other organisms.

The presence of urate oxidase also provides further support for peroxisomes in some lineages of Apicomplexa (Moog et al. 2017; Ludewig-Klingner et al. 2018), because urate oxidase activity is confined to peroxisomes in eukaryotes (Usuda et al. 1994). In addition to urate oxidase, *Nephromyces* and *Cardiosporidium* encode more peroxisome-associated proteins than *Plasmodium*, and nearly the same complement of genes encoded by *Toxoplasma* (table 2)*.* There are a few notable differences between *Toxoplasma* and *Nephromyces/Cardiosporidium*, including the absence of *PEX3, PEX16, VLACS, and SCPX* in *Nephromyces/Cardiosporidium* and the absence of *PMP22, GSTK1, DHRS4, XDH, and UO* in *Toxoplasma.* Additionally, *Nephromyces* encodes a copy of *MLS* absent in both *Cardiosporidium* and *Toxoplasma*. MLS is a key gene in the glyoxylate cycle, a pathway maintained in the photosynthetic *Chromera velia* and *Vitrella brassicaformis*, but lost in all other apicomplexans (Ludewig-Klingner et al. 2018). *Nephromyces/Cardiosporidium* also encode the enzyme serine-pyruvate transaminase (AGXT), which also uses glyoxylate as a substrate. AGXT converts glyoxylate into glycine and pyruvate and is often localized to peroxisomes; however, the localization of AGXT in *Nephromyces/Cardiosporidium* is unclear (supplementary table 1, Supplementary Material online).

**Table 2:** Peroxiomal Genes Identified in *Nephromyces* and *Cardiosporidium* and Their Functional Category

	Protein	Abbr.	Vb	Cv	C	N	Tg	Pf	Cp
Glyoxylate pathway									
	Isocitrate lyase	ICL	x	x	−	−	−	−	−
	MLS	MLS	x	x		x	−	−	−
	Citrate synthase	CS	x	x	x	x	x	x	−
	Aconitase	ACO	x	x	x	x	x	x	−
	Malate dehydrogenase	MDH	x	x	x	x	x	x	x
Peroxisome									
	Biogenesis factor 1	Pex1	x	x	−	x	x	−	−
	Biogenesis factor 2	Pex2	x	x	x	x	x	−	−
	Biogenesis factor 3	Pex3	x	x	−	−	x	−	−
	Ubiquitin carrier protein	Pex4	x	x	x	x	x	x	x
	Biogenesis protein 5	Pex5	x	x	x	x	x	−	−
	Biogenesis protein 6	Pex6	x	x	x	x	x	−	−
	Biogenesis protein 7	Pex7	x	x	x	x	x	−	−
	Biogenesis protein 10	Pex10	x	x	x	x	x	−	−
	Biogenesis factor 11	Pex11	x	x	x	x	x	−	−
	Biogenesis protein 12	Pex12	x	x	−	x	x	−	−
	Biogenesis factor 13	Pex13	−	−	−	−	−	−	−
	Membrane protein 14	Pex14	x	x	x	x	x	−	−
	Membrane protein 15	Pex15	−	−	−	−	−	−	−
	Biogenesis factor 16	Pex16	x	x	−	−	x	−	−
	Membrane protein receptor	Pex19	x	−	−	−	−	−	−
	Biogenesis protein 22	Pex22	x	x	x	x	x	x	x
	Biogenesis factor 26	Pex26	−	−	−	−	−	−	−
	Membrane channel	PMP22	x	x	x	x	−	−	−
	Membrane protein 4	PMP27	−	−	−	−	−	−	−
	ATP/ADP-transporter	PMP34	x	x	x	x	x	−	−
	Fatty acid ABC-transporter	PMP70	x	x	x	x	x	−	−
	ROS metabolism	MPV17	x	x	x	x	x	x	−
	Protein	Abbr.	Vb	Cv	C	N	Tg	Pf	Cp
Fatty acid oxidation									
α-Oxidation	2-Hydroxyacyl-CoA lyase	HPCL2	−	x	−	−	−	x	−
	Phytanoyl-CoA hydrolase	PHYH	x	x	−	−	−	−	−
β-Oxidation	α-Methylacyl-CoA-racemase	AMACR	−	−	−	−	−	−	−
	Acyl-CoA-oxidase	ACOX	x	x	x	x	x	−	−
	Multifunctional protein	DBP	x	x	x	x	x	−	−
	Sterole carrier protein 2	SCPX	−	x	−	−	x	−	−
	Multifunctional protein	PBE	−	−	−	−	−	−	−
	Acetyl-CoA acyltransferase 1	ACAA1	x	x	x	x	x	−	−
	2,4-dienoyl-CoA reductase	PDCR	x	x	x	x	x	−	−
	d(3,5)-d(2,4)-dienoyl-CoA isomerase	ECH	x	x	x	x	x	−	−
	ATP-binding cassette, subfamily D	ABCD	x	x	x	x	x	−	−
	Long-chain acyl-CoA synthetase	ACSL	x	x	x	x	x	x	−
	Solute carrier family 27, member 2	VLACS	x	x	−	−	x	−	−
Other oxidation	Acyl-CoA thioesterase 8	PTE	−	−	−	−	−	−	−
	Nucleoside disphosphate-linked m.	NUDT19	−	x	−	−	−	−	−
Amino acid metabolism							−	−	
	Multifunctional protein	AGT	x	x	x	x	x	−	−
	d-Amino-acid oxidase	DAO	−	−	−	−	−	−	−
	Isocitrate dehydrogenase	IDH	x	x	x	x	x	x	−
	*N*1-acetylpolyamine oxidase	PAOX	x	x	−	−	−	−	−
	l-Pipecolate oxidase	PIPOX	x	x	−	−	−	−	−
	Hydroxymethylgluatryl-CoA lyase	HMGCL	x	x	x	x	x	−	−
	(S)-2-hydroxy-acid oxidase	HAO	x	x	x	x	−	−	−
Antioxidant system							−	−	
Hydrogen peroxide metabolism	Catalase	CAT	x	−	x	x	x	−	−
	Superoxide dismutase	SOD	−	−	x	x	x	x	x
	Nitric-oxide synthase, inducible	INOS	−	−	−	−	−	−	−
	Peroxiredoxin 1	PRDX1	x	x	−	x	x	x	x
	Peroxiredoxin 5	PRDX5	−	−	−	−	−	−	−
Glutathione metabolism									
	Glutathione S-transferase kappa 1	GSTK1	x	x	x	x	−	−	−
	Protein	Abbr.	Vb	Cv	C	N	Tg	Pf	Cp
Etherphospholipid biosynthesis									
	Dihydroxyacetone phosphate acyltr.	DHAPAT	x	x	x	x	x	−	−
	Alkyldihydroxyacetone phosphate syn	AGPS	x	x	x	−	−	−	−
	Fatty acyl-CoA reductase	FAR	x	x	−	x	x	−	−
Purine metabolism									
	Xanthine dehydrogenase	XDH	x	x	x	x	−	−	−
	Urate oxidase	UO	x	x	x	x	−	−	−
Retinol metabolism									
	Dehydrogenase/reductase SDR family	DHRS4	x	x	x	x	−	−	−
Sterol precursor biosynthesis									
	Mevalonate kinase	MVK	−	−	−	−	−	−	−
	Phosphomevalonate kinase	PMVK	−	−	−	−	−	−	−

Note.—(x) denotes presence of gene and (−) absence. Vb, *Vitrella brassicaformis*; Cv, *Chromera velia*; C, *Cardiosporidium*; N, *Nephromyces*; Tg, *Toxoplasma gondii*; Pf, *Plasmodium falciparum*; Cp, *Cryptosporidium parvum*. Table modified based on Ludwig-Klinger et al. (2017).

## Discussion

The recent scrutiny by Moog et al. (2017) and Ludewig-Klingner et al. (2018) has built a case for the presence of peroxisomes in some apicomplexan lineages. While some apicomplexans may have lost peroxisomes, it seems likely that it is not a universally shared trait in the phylum. Despite the extensive search for peroxisome-associated functions in apicomplexans, no genes involved in purine degradation were found in other sequenced apicomplexan genomes, with the lone exception of allantoicase in *Plasmodium* (Gardner et al. 2002). Our in silico predictions indicate a complete purine degradation pathway in *Nephromyces* and *Cardiosporidium.* In addition to highly expressed transcripts for the genes involved, all of the identified purine degradation genes and *MLS* have been located on genomic contigs from *Nephromyces*. Based on neighboring genes and the presence of introns in the *Nephromyces* genes matching the expressed transcripts, these contigs almost certainly originate from the *Nephromyces* genome (table 1). Additionally, none of the purine degradation transcripts attributed to *Nephromyces* was detected in uninfected tunicates (table 3). Phylogenetic trees of purine degradation genes are poorly supported at an interphylum level, indicating a rapid evolutionary rate. Although most genes are phylogenetically uninformative across the spectrum of eukaryotes, these gene trees have strong support for monophyly of purine degradation genes from *Nephromyces* and *Cardiosporidium* with Chromerids (fig. 1). The combination of gene trees, expression only when *Nephromyces* is present, and preliminary genomic assemblies strongly suggests that these genes were present since the divergence of Apicomplexa and Chromerida and have been vertically transmitted. Thus, these genes have been subsequently lost across apicomplexans, possibly multiple times. Although the exact placement of *Nephromyces* and *Cardiosporidium* is not certain (Saffo et al. 2010), multigene phylogenies place them in the subclass Hematozoa (Muñoz et al., in preparation), suggesting that purine degradation was independently lost multiple times in Apicomplexa as well as maintained long after apicomplexans had become obligate parasites.

**Table 3:** Expression Percentile Ranking of Purine Degradation Genes, from Total Expressed Transcripts in *Nephromyces* (Neph), *Cardiosporidium* (Cardio), and *Molgula* (Mm)

Gene	Wild Neph	Lab-Grown Neph 1	Lab-Grown Neph 2	Cardio Fraction 1	Cardio Fraction 2	Cardio Fraction 3	Mm	Uninfected Mm 1	Uninfected Mm 2	Uninfected Mm 3
Xanthine dehydrogenase	97.87	93.17	94.83	none	76.88	69.5	93.64	N/A	N/A	N/A
Urate oxidase	99.87	99.44	99.54	86.75	87.24	70.98	−	−	−	−
5-Hydroxyisourate hydrolase	99.16	91.31	88.41	87.67	83.27	79.1	−	−	−	−
OHCU decarboxylase	93.38	−	−	−	−	−	−	−	−	−
Allantoinase	99.09	98.38	98.23	73.61	90.32	71.89	−	−	−	−
Aminodohydrolase	99.75	79.25	89.18	87.43	92.27	92.08	−	−	−	−
MLS	59.17	93.81	93.11	−	−	−	−	−	−	−
AGXT	99.85	99.57	99.79	84.64	80.81	77.79	85.65	91.17	71.85	75.05

Note.—The wild *Nephromyces* and *Molgula manhattensis* data originate from the same RNA extraction and were bioinformatically separated. Data were also generated from lab-grown tunicates, artificially infected with *Nephromyces* (Lab-Grown Neph 1 and 2). *Cardiosporidium* fractions represent 1) unfiltered pericardial fluid, 2) the 25% and 3) 30% fractions extracted from a sucrose gradient, and may contain different proportions of *Cardiosporidium* life stages. The three uninfected *Molgula manhattensis* were raised from gametes in the lab and never exposed to *Nephromyces* infection. The (−) denotes the transcript was not recovered in that data set, whereas (N/A) indicates the transcript was assembled, but the transcripts per million was <1.

The presence of predicted purine degradation genes in *Nephromyces* and *Cardiosporidium* adds a function not previously demonstrated in apicomplexan peroxisomes (table 2; Moog et al. 2017; Ludewig-Klingner et al. 2018). While *Toxoplasma* and *Cardiosporidium/Nephromyces* share many of the same peroxisomal marker genes, no copy of *PEX3* has been found in *Cardiosporidium/Nephromyces.PEX3* (along with *PEX10, PEX12, and PEX19*) is one of the four genes reportedly required for peroxisome function (Schluter et al. 2006). However, the fundamentals of peroxisome biology have been described from a limited set of eukaryotes, and organisms such as ciliates have peroxisomes and lack PEX3 (Ludewig-Klingner et al. 2018). Therefore, PEX3 may not be critical to peroxisome function for alveolates, and possibly other understudied eukaryotic lineages. Extreme sequence conservation of PEX3 and PEX19 is only found in opisthokonts, and sequence divergence in other lineages may indicate alternative functions (Hua et al. 2015).

Two other genes (*Sterol carrier protein 2, SCPX and Solute carrier family 27, member 2, VLACS*) missing from *Cardiosporidium/Nephromyces*, but found in *Toxoplasma*, are involved in β*-*fatty acid oxidation. Both *Cardiosporidium/Nephromyces* encode the seven other β*-*fatty acid oxidation genes encoded in *Toxoplasma*, suggesting β*-*fatty acid oxidation forms part of the functional capabilities of the *Cardiosporidium/Nephromyces* peroxisome*.* Fatty acid oxidation is often a central component of peroxisome function and has been hypothesized to be the impetus for the evolution of peroxisomes (Speijer 2011).

Based on transcript abundance, purine degradation in *Nephromyces* peroxisomes appears to be heavily utilized. Only 0.13% of genes had a higher transcription rate than urate oxidase in our data from wild collected *Nephromyces*, and the other genes in the purine degradation pathway are among the most highly expressed transcripts in both wild and lab-grown *Nephromyces* samples (table 3). This result aligns with the previously reported high levels of urate oxidase protein in the renal sac of infected *Molgula* (Saffo 1988), indicating that the expression levels reported here do translate to protein. Much of this pathway is expressed over the 99th percentile of all transcripts in *Nephromyces*, which corresponds to the top 100 genes. Expression of purine degradation genes in *Cardiosporidium* is far lower, and in the 70–90 percentile range (table 3). Such high expression in *Nephromyces* represents an enormous metabolic investment, and it is unlikely that these transcripts go largely untranslated.

Both *Nephromyces* and *Molgula manhattensis* encode xanthine dehydrogenase, and are able to convert xanthine into uric acid. As we have identified the tunicate host as the source of purines, this raises the question of why *Nephromyces* is expressing xanthine dehydrogenase in the 97.87th percentile, compared with similarly high tunicate expression (93.64th percentile). Although the percentile ranking between these two organisms cannot be directly compared, such high xanthine dehydrogenase expression in *Nephromyces* is surprising. It seems unlikely that so much xanthine dehydrogenase production is needed to convert only endogenous purines of *Nephromyces*. However, xanthine is only detected in the renal sac in small quantities, not nearly as abundant as uric acid, and xanthine dehydrogenase activity is restricted to the renal wall, not the renal lumen (Nolfi 1970). One possible explanation is that *Nephromyces* exports its xanthine dehydrogenase into the renal wall in order to drive the production of xanthine from hypoxanthine before the purine salvage enzymes adenine phosphoribosyltransferase and hypoxanthine–guanine phosphoribosyltransferase can salvage hypoxanthine into adenine and guanine.

High expression of purine degradation genes in *Nephromyces* is clear, but the purpose is uncertain. It does indicate purine degradation is an important pathway for *Nephromyces*; however, the functional significance is not immediately obvious. Pathway analysis predicts that *Nephromyces* is able to convert xanthine into urea and ureidoglycolate; however, neither compound is biologically useful without further conversion. We propose that the products of purine degradation in *Nephromyces* are converted to glyoxylate.

One possible route is the conversion of ureidoglycolate into glyoxylate. There are two known enzymes able to catalyze this conversion: ureidoglycolate lyase, found in fungi and bacteria, which catalyzes (s)-ureidoglycolate to glyoxylate and urea, and ureidoglycolate amidohydrolase, found in plants and bacteria, which catalyzes (s)-ureidoglycolate to glyoxylate, carbon dioxide, and ammonia (Wells and Lees 1991; Muñoz et al. 2006; Serventi et al. 2010; Werner et al. 2010; Shin et al. 2012; Percudani et al. 2013). Both ureidoglycolate lyase and ureidoglycolate amidohydrolase are amidohydrolases—hydrolases that use amide bonds as substrates. No orthologs to either ureidoglycolate lyase or ureidoglycolate amidohydrolase have been found in the *Nephromyces* transcriptome. However, an amidohydrolase is present, which is predicted to be structurally similar to the ureidoglycolate amidohydrolase found in *Arabidopsis*, including similar location and number of zinc-binding domains*.* This amidohydrolase also has a similarly high expression level as the other purine degradation enzymes (table 3). In order to determine whether the amidohydrolase found in *Nephromyces* is capable of catalyzing (s)-ureidoglycolate, functional assays will need to be performed.

While the functionality of this particular amidohydrolase has yet to be determined, its ability to act on an (s)-ureidoglycolate is an attractive hypothesis for a few reasons. One, there are two known enzymes capable of breaking the amide bond in (s)-ureidoglycolate that have independently evolved: ureidoglycolate lyase and ureidoglycolate amidohydrolase. This pathway has not been widely explored across eukaryotes, and the modification to a class of molecules able to break amide bonds to accommodate the structure of (s)-ureidoglycolate may not be a complex evolutionary step. Two, (s)-ureidoglycolate is unstable and will spontaneously convert to glyoxylate, albeit without the stereospecific conversion present when catalyzed by ureidoglycolate amidohydrolase (Werner et al. 2010). Spontaneous conversion of glyoxylate results in a 50% loss of efficiency versus enzymatic conversion, presumably creating strong evolutionary pressure to enzymatically degrade (s)-ureidoglycolate to maintain stereochemistry.

Glyoxylate is a common substrate for a number of enzymes including glyoxylate oxidase, which catalyzes glyoxylate with water and oxygen to form oxalate and hydrogen peroxide (Kasai et al. 1963). Notably, no copy of glyoxylate oxidase has been identified in *Nephromyces*, which is surprising given that another common component of the renal sac is calcium oxalate (Saffo and Lowenstam 1978). We have not identified any genes suggesting that *Nephromyces* or its bacterial endosymbionts can produce or process oxalate, oxalate is also found in uninfected hosts indicating that the source of the calcium oxalate is likely the tunicate. Another enzyme that uses glyoxylate as a substrate, which is present in *Nephromyces/Cardiosporidium*, is AGXT, which can be localized to peroxisomes or mitochondria, and catalyzes glyoxylate to glycine and pyruvate (Takada and Noguchi 1985). An alternative enzyme for processing glyoxylate is MLS, which is also targeted to the peroxisome and missing from apicomplexans, including *Cardiosporidium*, but is found in *Nephromyces* (fig. 1).

MLS is one of two genes integral to the glyoxylate cycle, an alternative pathway for part of the citrate cycle. In the glyoxylate cycle, isocitrate is converted into glyoxylate and succinate by isocitrate lyase (McFadden and Howes 1965). Glyoxylate is combined with acetyl-CoA to create malate (Molina et al. 1994). This cycle allows for the creation of glucose from fatty acids directly (Kornberg and Krebs 1957). The presence of MLS indicates at least a piece of the glyoxylate cycle is present in *Nephromyces*. No copy of isocitrate lyase is predicted from the *Nephromyces* transcriptome, and only a small fragment of a possible isocitrate synthase has been identified in *Cardiosporidium*. However, under the model proposed here, the generation of glyoxylate is from uric acid, and isocitrate synthase would not be required.

**Fig. 2.**
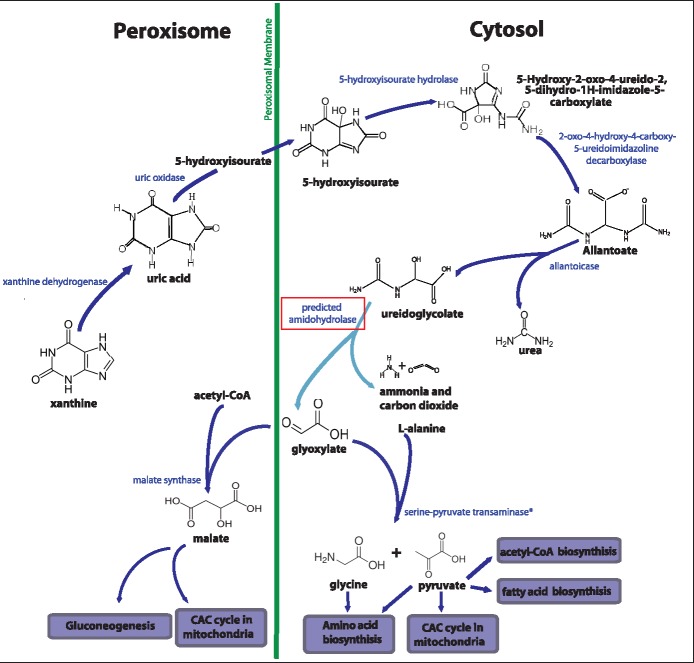
—Predicted purine degradation pathway in *Nephromyces*, within the peroxisome and cytosol. Dark blue arrows represent enzymes identified in the *Nephromyces* transcriptome. The light blue arrow represents the highly expressed amidohydrolase (red box) predicted to convert ureidoglycolate into glyoxylate. Enzymes on the left side are localized to peroxisomes, the right side to the cytosol, with the green vertical line representing the peroxisomal membrane. The predicted pathway is able to convert uric acid into glyoxylate, and subsequent conversion by AGXT or MLS, creates glycine and pyruvate or malate, respectively. The * by AGXT indicates ambiguous predicted localization, to either peroxisomes or mitochondria.

Both AGXT and MLS (in *Nephromyces*) show similarly high expression as the purine degradation genes (table 3), which is consistent with our proposed uric acid to glyoxylate pathway. In particular, AGXT is among the most highly expressed *Nephromyces* transcripts, with consistently higher expression than MLS, possibly indicating it is the primary route of glyoxylate conversion. The products of AGXT, glycine and pyruvate, are versatile substrates and used by a number of pathways. Glycine is the simplest amino acid and an essential component of many important biological compounds, as a nitrogen source in a readily useable form. Pyruvate is extremely versatile and involved in several critical biological pathways. A noninclusive list includes amino acid biosynthesis, acetal-CoA biosynthesis, fatty acid biosynthesis, and the citric acid cycle. These pathways represent both carbon and energy acquisition (fig. 2). Additionally, *Nephromyces* has the ability to use MLS to convert glyoxylate and acetal-CoA into malate, a compound central to the citric acid cycle, allowing for another mechanism of carbon and energy acquisition (fig. 2).

The hypothesized conversion of uric acid to glyoxylate in *Nephromyces* creates several possibilities. First, it allows for the metabolic waste product, uric acid, to be converted into glycine, pyruvate, and malate (fig. 2). Second, it provides an explanation for the exceptionally high expression of the purine degradation pathway. Third, it gives *Nephromyces* access to a primary carbon, nitrogen, and an energy source at no cost to its host. And finally, this change in primary carbon, nitrogen, and energy could conceivably reduce the impact of *Nephromyces* on its host, allowing *Nephromyces* densities to increase while decreasing virulence. Reduction in virulence would have been a necessary first step toward mutualism.

Uric acid as a primary carbon and energy source is not completely unknown. Bacterial species have been found in chicken hutches that were able to grow solely on uric acid (Rouf and Lomprey 1968; Thong-On et al. 2012), and some species of fungi are able to grow on media solely containing uric acid (Middelhoven et al. 1989). However, this is a novel substrate for an apicomplexan to grow on, and while it is unlikely that *Nephromyces* could survive on uric acid alone, it is a promising base for both carbon and nitrogen acquisition. It is possible that the *Nephromyces* bacterial endosymbionts (Potrikus and Breznak 1980; Sabree et al. 2009) are contributing to the proposed purine to glucose pathway, but that is not currently supported by our data.

As the adaptive significance of uric acid deposits in tunicates, and particularly in *Molgula*, is unknown, it is difficult to speculate on the effects of *Nephromyces* uric acid degradation to the host. If these renal sac deposits are a form of excretion by storage, as has been hypothesized (Goodbody 1965), then having a symbiont that is capable of digesting uric acid may be beneficial simply by digesting an indigestible metabolite and converting uric acid into urea. Alternatively, once the uric acid has been broken down, the tunicate may benefit from metabolites derived from uric acid previously unavailable to the tunicate. If *Nephromyces* is overexpressing xanthine dehydrogenase in order to outcompete adenine phosphoribosyltransferase and hypoxanthine–guanine phosphoribosyltransferase, diverting hypoxanthine from purine salvage to purine degradation, there could be a potential cost to the host under purine-limited conditions.

Our data demonstrate that both the proposed mutualistic *Nephromyces* and parasitic *Cardiosporidium* encode the genes for purine degradation, which have been lost in other apicomplexans, sequenced to date. Additionally, these genes share a common ancestry with chromerid genes, indicating they are not the product of a recent horizontal gene transfer from bacteria. These data also add support to the growing body of evidence that indicate the presence of peroxisomes in apicomplexans. *Nephromyces* and *Cardiosporidium* are predicted to have peroxisomes and, unlike any other apicomplexan, are capable of preforming both purine degradation and part of the glyoxylate cycle. The presence of purine degradation, AGXT, and MLS allow for the intriguing possibility of conversion of uric acid into a primary nitrogen, carbon, and energy source. This predicted metabolic activity would be a completely novel substrate for an apicomplexan and may have been an important factor in the reduction of virulence in *Nephromyces*.

## Supplementary Material

Supplementary data are available at *Genome Biology and Evolution* online.

## Supplementary Material

Supplementary Data
